# Appetite regulating genes may contribute to herbivory versus carnivory trophic divergence in haplochromine cichlids

**DOI:** 10.7717/peerj.8375

**Published:** 2020-01-20

**Authors:** Ehsan P. Ahi, Anna Duenser, Pooja Singh, Wolfgang Gessl, Christian Sturmbauer

**Affiliations:** 1 Evolutionary Biology Centre, Uppsala University, Uppsala, Sweden; 2 Institute of Biology, University of Graz, Graz, Austria; 3 Institute of Biological Sciences, University of Calgary, Calgary, Canada

**Keywords:** Appetite regulation, East African Lakes, Cichlids, Gene expression, Trophic specialization, Adaptive radiation, Anorexigenic, Orexigenic, Brain, Larval development

## Abstract

Feeding is a complex behaviour comprised of satiety control, foraging, ingestion and subsequent digestion. Cichlids from the East African Great Lakes are renowned for their diverse trophic specializations, largely predicated on highly variable jaw morphologies. Thus, most research has focused on dissecting the genetic, morphological and regulatory basis of jaw and teeth development in these species. Here for the first time we explore another aspect of feeding, the regulation of appetite related genes that are expressed in the brain and control satiety in cichlid fishes. Using qPCR analysis, we first validate stably expressed reference genes in the brain of six haplochromine cichlid species at the end of larval development prior to foraging. We next evaluate the expression of 16 appetite related genes in herbivorous and carnivorous species from the parallel radiations of Lake Tanganyika, Malawi and Victoria. Interestingly, we find increased expression of two appetite-regulating genes (anorexigenic genes), *cart* and *npy2r,* in the brain of carnivorous species in all the three lakes. This supports the notion that appetite gene regulation might play a part in determining trophic niche specialization in divergent cichlid species, already prior to exposure to different diets. Our study contributes to the limited body of knowledge on the neurological circuitry that controls feeding transitions and adaptations in cichlids and other teleosts.

## Background

At the onset of exogenous feeding, most marine and freshwater fish species are known to be planktivorous and have a limited ability to detect, capture, ingest and digest prey (Nunn, Tewson & Cowx, 2012). Improvements in vision, swimming performance, gaping abilities and the functions of digestive tract during ontogeny can lead to shifts in diet composition and preference. Importantly, differences in feeding behaviour between fish species both at early stages of exogenous feeding and during later ontogeny can influence diet preference at inter- and intraspecific levels (Nunn, Tewson & Cowx, 2012). Little is known about the role of central nervous system in determining the diet preference and related feeding behaviours required for using specific food resources in natural ecosystems. Repeated evolution of herbivorous and carnivorous trophic specialization in closely related teleost fishes is a prominent example for such a divergence in diet preference, and so far, most studies were mainly focused on related changes in digestive system (morphological and enzymatic differences) (Kapoor, Smit & Verighina, 1976; Chakrabarti et al., 1995; Chan et al., 2004; German et al., 2010). Besides morphological changes in the feeding apparatus, divergence of feeding behaviour might be another key feature of differential trophic adaptation since the two diet habits require foraging on different quantities of food to balance nutritional requirements due to the unequal quality of these diets. Since adult herbivorous and carnivorous fish have distinct appetite and feeding behaviours, these differences might be pre-determined or influenced by early changes in activity of genes controlling feeding behaviours at the onset or prior to exogenous feeding. In the latter case, one would expect to observe differences in expression of these genes at the end of the larval stage, which reflects the feeding behaviour of the adults, by differentiating herbivorous and carnivorous species.

One of the major events preceding differences in feeding behaviour can be changes in activity of appetite-regulating genes in the central nervous system, which later translate into distinct desires for food intake and related locomotory activities (e.g., food searching) (Volkoff et al., 2005). Thus, an immediate approach to investigate such molecular mechanisms is expression analysis of genes involved in regulation of feeding behaviour through the central nervous system in fish (Volkoff et al., 2005). To date, only one study in grass carp (a species of Cypriniformes), which shows the transition from carnivory to herbivory during ontogeny, has addressed gene expression changes in the brain between the two contrasting feeding habits (He et al., 2015). Interestingly, the authors found that few appetite-regulating genes that inhibit food intake (anorexigenic genes) had reduced expression in the brain at the herbivorous life stage, whereas few other genes with opposite effects (orexigenic genes) had increased expression at this stage (He et al., 2015). This finding was consistent with the notion that herbivory requires prolonged insatiety and more active feeding behaviour compared to carnivory in order to compensate for the relatively poorer nutritional quality of a plant-based diet (He et al., 2015). Although, a comprehensive list of potential appetite-regulating genes has been provided mainly from studies on cyprinid model species, such as zebrafish and goldfish, it has turned out that the regulatory function of many of these genes can vary across the orders of teleost fishes (Volkoff, 2016). In addition, only a small subset of the genes are confirmed to have similar appetite-regulating functions in other fish orders including Cichliformes and Perciformes (Volkoff, 2016).

Cichlids of the East African Great Lakes Tanganyika, Malawi and Victoria are well known for their remarkable rates of speciation and adaptive radiation (Fryer & Iles, 1972; Kocher, 2004). Lake Tanganyika, the oldest of the three lakes, shows the most diversity in ecomorphology, behaviour and genetics compared to Lake Malawi, the intermediate, and Lake Victoria, the youngest of the three lakes (Young, Snoeks & Seehausen, 2009; Salzburger, Van Bocxlaer & Cohen, 2014). The Haplochromini are the most species rich tribe, having seeded the entire species flocks of Lake Malawi and Victoria and also recolonized Lake Tanganyika, giving rise to the tribe Tropheini (Salzburger et al., 2005). It is hypothesized that similar trophic ecomorphologies evolved in all three lakes in response to similar selection pressures as they were derived from a common generalist riverine ancestor (Kocher et al., 1993; Salzburger et al., 2005; Cooper et al., 2010).

Interestingly, haplochromine cichlids are mostly maternal mouthbrooders so the fry start feeding independently at a more mature stage, at the end of larval development (stage 26), compared to non-mouthbrooders (Fujimura & Okada, 2007; Fujimura & Okada, 2008). At stage 26, the larvae leave the buccal cavity of the mother and are exposed for the first time to the environment as they start foraging independently. Due to the high trophic phenotypic plasticity in haplochromine cichlids (Gunter et al., 2013; Schneider et al., 2014), it is important to decipher whether gene regulatory circuitry of appetite-regulating genes that triggers feeding behaviour can be already observed upon completion of the larval development prior to the onset of food intake or is activated once the larvae start feeding. The dietary plasticity, mouthbrooding and swimming behaviours (e.g., locomotory activities), and immense diversity of trophic specializations and foraging in cichlid fishes of East African species flocks provide an excellent opportunity to investigate the role of appetite-regulating genes in differential trophic adaptations associated with species divergence.

Here, we hypothesize that appetite-regulating genes might be already differentially regulated in the brain of distinctly adapted haplochromine cichlids at the end of larval development, thus, affecting feeding behaviour (i.e., food intake and related locomotory activity), before the fry is released from the mother’s mouth to forage on their own. Our hypothesis might advocate for low plasticity and high genetic wiring of feeding behaviour in these fish, although studies investigating potential effects of appetite-regulating genes on foraging tactics are required to make such a claim. Therefore, we selected 16 appetite-regulating genes and analysed their expression level in the brain in a set of three herbivorous and three carnivorous haplochromine cichlid fish species at stage 26 (Fujimura & Okada, 2007; Fujimura & Okada, 2008), which marks the end of larval development and the initiation of exogenous feeding. The selected candidate genes are known to have brain expression in fish and are involved in regulation of feeding behaviour by enhancing or inhibiting food intake in teleost fishes (Table 1). The functions of most of these genes have been investigated in Cypriniformes (on which most studies have been conducted) and fish species with evolutionary closer relatedness than Cypriniformes to cichlids, such as members of Perciformes and Cichliformes. Five of these genes are also playing a role in food habit transition from carnivory to herbivory in grass carp (He et al., 2015) (Table 1). The study species belong to two major trophic niches in the three Great East African Lakes; Lake Tanganyika (LT), Lake Malawi (LM) and Lake Victoria (LV). We test whether the differential expression of appetite-regulating genes in the brain predicts the divergence in trophic specialization in differentially adapted species pairs prior to the actual searching for food resources. The study also addresses this possibility in the context of parallel trophic specialization across three independent adaptive radiations. This study reports the results of a first step by validation of stably expressed reference genes in the brain at the end of the larval stage, which allows us to accurately compare inter-species expression of the appetite regulating-genes in haplochromine cichlids. Our results suggest that expression differences of the candidate genes might predict the feeding behaviour of herbivore versus carnivore species before the onset of plastic molecular responses emanating from contrasting feeding diets.

**Table 1:** Selected appetite-regulating genes in this study.

**Gene**	**Description**	**Organisms**	**Effects**	References
*agrp2*	Agouti related neuropeptide 2	Perciformes Cypriniformes	Orexigenic Diet transition	Agulleiro et al. (2014), He et al. (2015)
*apln*	Apelin, agtrl1 Ligand	Perciformes Cypriniformes	Orexigenic	Hayes & Volkoff (2014), Volkoff (2016)
*cart*	Cocaine and amphetamine regulated transcript	Cichliformes Perciformes Cypriniformes	Anorexigenic	Babichuk & Volkoff (2013), Volkoff (2016), Porter, Roberts & Maruska (2017)
*cck*	Cholecystokinin triacontatriapeptide	Cichliformes Perciformes Cypriniformes	Anorexigenic	Grone et al. (2012), Babichuk & Volkoff (2013), Volkoff (2016)
*crh*	Corticotropin-releasing hormone	Salmoniformes Cypriniformes	Anorexigenic	Bernier & Craig (2005), Volkoff (2016)
*drd1*	Dopamine receptor D1	Cypriniformes	Anorexigenic Diet transition	He et al. (2015)
*gabra1*	Gamma-aminobutyric acid A receptor alpha-1	Cypriniformes	Orexigenic Diet transition	Trudeau, Sloley & Peter (1993), Matsuda et al. (2011), He et al. (2015)
*hcrt*	Orexin, hypocretin neuropeptide precursor	Cichliformes Perciformes Cypriniformes	Orexigenic	Yan et al. (2011), Grone et al. (2012), Volkoff (2016)
*nmu*	Neuromedin U preproprotein	Perciformes Cypriniformes	Anorexigenic	Kono et al. (2012), Li et al. (2015), Volkoff (2016)
*npy*	Prepro-neuropeptide Y	Cichliformes Perciformes Cypriniformes	Anorexigenic ^?^ Orexigenic	Grone et al. (2012), Matsuda et al. (2012), Babichuk & Volkoff (2013), Volkoff (2016), Das et al. (2019)
*npy2r*	Neuropeptide Y receptor type 2	Perciformes Cypriniformes	Anorexigenic Diet transition	Matsuda et al. (2012), Wang et al. (2014), He et al. (2015)
*pacap*	Pituitary adenylate cyclase activating polypeptide	Cichliformes Cypriniformes	Anorexigenic	Matsuda et al. (2005), Zhou et al. (2013), Costa et al. (2016)
*pomc*	Pro-opiomelanocortin preproprotein	Cichliformes Cypriniformes	Anorexigenic	Volkoff (2016), Porter, Roberts & Maruska (2017)
*pyy*	Prepro-peptide YY	Perciformes Cypriniformes	Orexigenic Anorexigenic	Murashita et al. (2006), Volkoff (2016)
*trh,* *trhra*	Thyrotropin-releasing hormone and its receptor	Cypriniformes	Orexigenic Diet transition	He et al. (2015), Volkoff (2016)

## Methods

### Fish husbandry and sampling

**Figure 1:**
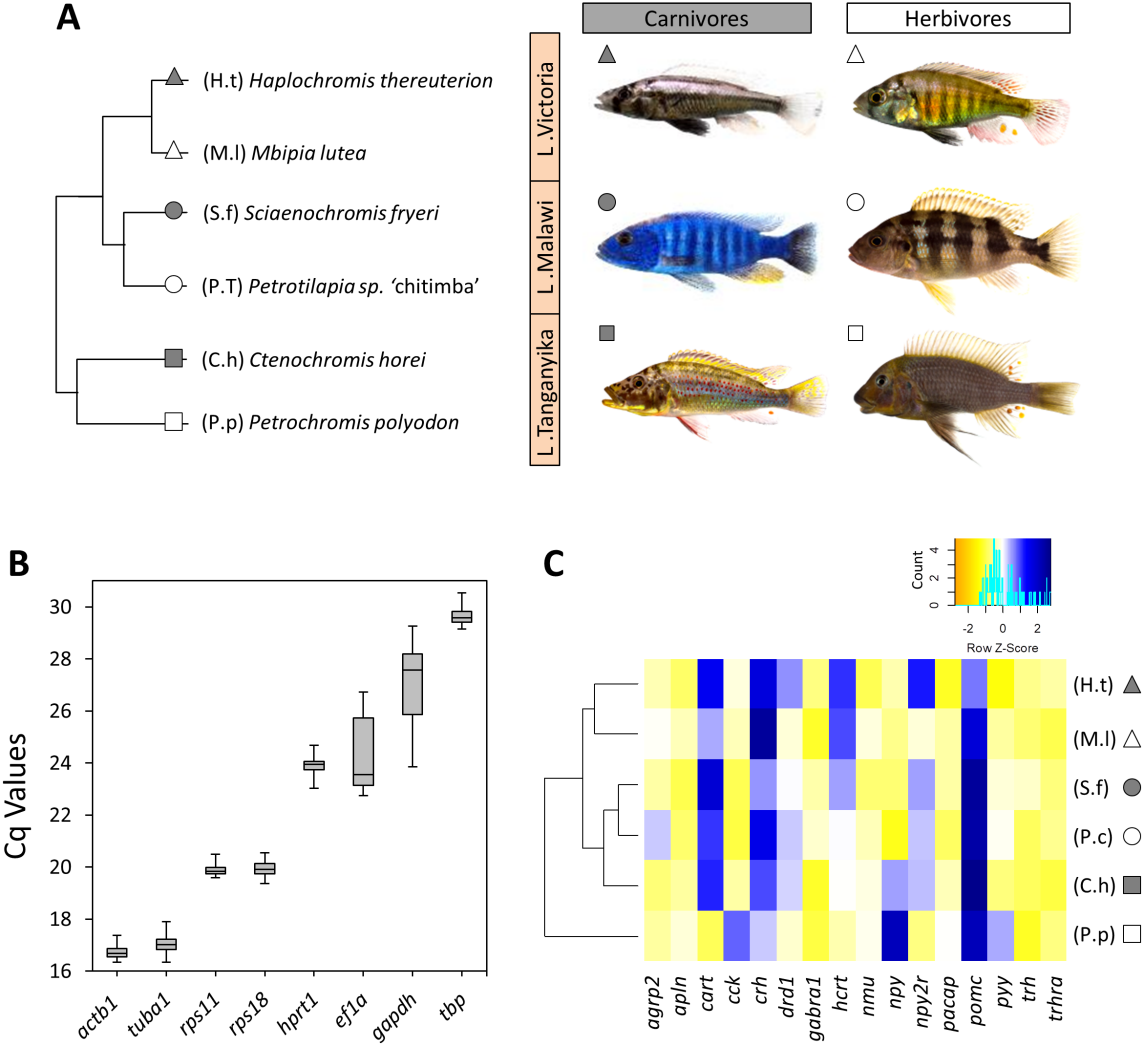
The haplochromine cichlid species in this study, expression levels of the reference genes and a hierarchical clustering based on expression pattern of appetite-regulating genes in the brains. (A) A simplified phylogenetic tree of the six East African haplochromine cichlids representing their relatedness specified by inhabiting lakes and trophic specializations. The colour of the symbol beside each species indicates trophic niche and its shape refers to inhabiting lake (cichlid fish photography by Wolfgang Gessl). (B) Expression levels of a selected set of reference genes using their Cq values in brain across the species. The middle line in each box plot represents the median together with the 25/75 percentiles. (C) A dendrogram clustering of species based similarity in expression levels of 16 appetite regulating genes in larval brain prior to foraging.

Six haplochromine cichlid species belonging to two major trophic niches from Lakes Tanganyika (LT), Malawi (LM) and Victoria (LV), were chosen for studying brain gene expression. All samples were obtained from lab stocks, which have been previously acquired from aquarium trade suppliers. In order to compare divergent trophic niches, we used one carnivorous species (a piscivore/insectivore) and one herbivorous species (an algae-grazer) for each lake (Fig. 1A), based upon previous phylogenetic studies (Koblmüller et al., 2008; Irissari et al., 2018). The parental fish were reared under standardized aquarium conditions and diet (Spirulina flakes with average protein content) until sexual maturation. The spawning pairs were closely observed and 24 h after mating their eggs were collected from the mouth of the females through exerting mild manual pressure to their cheeks. Then, the eggs of each species were placed in a standard glass jar with constant gentle shaking during the incubation period until hatching stage. After hatching, larvae were transferred to small floating tanks and kept until stage 26, the time of yolk sac absorption, marking the end of larval development (Fujimura & Okada, 2007; Fujimura & Okada, 2008). The rearing and incubation temperature was kept constant at 25.8 degrees centigrade. For each species six larvae were euthanized in water containing 0.2 gram MS-222 per litre, and the entire brain was carefully dissected using a stereomicroscope. The brain tissue from each individual represents one biological replicate, and therefore, six biological replicates per species were used for further analysis of gene expression. Moreover, by the end of the study the parents of the six haplochromine species were sacrificed in water containing 0.8 gram MS-222/litre.

### RNA isolation and cDNA synthesis

The entire brain from each individual was dissected as one biological replicate and transferred into a tube with 250 µL of a lysis buffer, specific for RNA isolation from tissue, provided by Reliaprep RNA tissue miniprep system (Promega, #Z6111, USA). A 1.4 mm ceramic bead was added to shred the brain tissue. The brains were homogenized in a FastPrep-24 Instrument (MP Biomedicals, Santa Ana, CA, USA) and total RNA content was extracted following the manufacturer protocol. The protocol has several relatively quick steps; mixing of the homogenized tissue (in the lysis buffer) with isopropyl alcohol and filtering it through a column provided by the kit, RNA washings and gDNA removal. Next, the RNA quantity was measured using a Nanophotometer (IMPLEN GmbH, Munich, Germany) and the quality was evaluated with RNA ScreenTapes on an Agilent 2200 TapeStation (Agilent Technologies). The extracted RNAs with a RIN (RNA integrity number) above seven were used for first strand cDNA synthesis using 500ng total RNA input and High Capacity cDNA Reverse Transcription kit (Applied Biosystems). The cDNAs were diluted 1:10 times in RNase-free water in order to proceed with qPCR. It is worth emphasizing that the Reliaprep RNA kit was successful in extracting high quality RNA from brain tissue regardless of the high level of fat content, thus the kit can be recommend for RNA extraction from other fatty tissues (e.g., oocyte and adipose tissues (Lecaudey et al., in press; Ahi et al., 2018) for which the conventional methods might not yield acceptable RNA quality for gene expression studies.

### Gene selection and primer design

We selected a non-exhaustive list of 16 appetite-regulating genes that are studied in teleost fish, of which five genes play a role in food habit transition from carnivory to herbivory in grass carp (Table 1). In order to precisely measure the expression of the appetite-regulating genes in the brain, identification of stably expressed reference genes with minimum expression variation among the samples is considered as first crucial step in qPCR (Kubista et al., 2006). Therefore we chose eight candidate genes that are found to be among stably expressed reference genes in qPCR studies across different tissues in East African cichlids (Yang et al., 2013; Gunter & Meyer, 2014; Ahi & Sefc, 2017a; Ahi & Sefc, 2017b); (Ahi, Richter & Sefc, 2017; Ahi et al., 2019a; Ahi et al., 2019b). To design primers, we used conserved coding sequence regions based on the transcriptomes of several East African haplochromine species (*Pundamilia nyererei*, *Simochromis diagramma*, *Gnathochromis pfefferi*, *Metriaclima zebra*, and *Astatotilapia burtoni*) and two other cichlid species belonging to distant tribes (*Oreochromis niloticus* and *Neolamprologus brichardi*) (Brawand et al., 2014; Singh et al., 2017). The sequences from all species were first aligned in CLC Genomic Workbench, version 7.5 (CLC Bio, Aarhus, Denmark) and exon/exon junctions were identified through the annotated genome of *Astatotilapia burtoni* in the Ensembl database (http://www.ensembl.org) (Zerbino et al., 2018). The designed primers were spanning the exon/exon with small amplicon size (<200 bp) which is optimal for qPCR quantification (Fleige & Pfaffl, 2006). We used OligoAnalyzer V3.1 software (http://www.idtdna.com/analyzer/Applications/OligoAnalyzer) and Primer Express V3.0 (Applied Biosystems, CA, USA) to design the primers with minimal dimerization and secondary structures.

### qPCR and expression data analysis

In order to prepare qPCR reactions, we followed the protocol suggested by the manufacturer; Maxima SYBR Green/ROX qPCR Master Mix (2X) (Thermo Fisher Scientific, Germany). The qPCR amplifications were conducted in 96 well-PCR plates using ABI 7500 real-time PCR System (Applied Biosystems) with two technical replicates for each biological replicate and observing the experimental set-up known as sample maximization method to attain optimal qPCR conditions (Hellemans et al., 2007). The qPCR program and a dissociation step were performed as described in a previous gene expression study of cichlids (Ahi & Sefc, 2018), and the amplification efficiency of each primer pair was calculated through LinRegPCR v11.0 programme (http://LinRegPCR.nl) (Ramakers et al., 2003) (Table S1).

Three common algorithms for reference validation, BestKeeper (Pfaffl et al., 2004), NormFinder (Andersen, Jensen & , 2004) and geNorm (Vandesompele et al., 2002) were used to rank the most stably expressed reference genes. BestKeeper calculate an index which considers the lowest standard deviations (SD) of Cq values for its ranking, whereas geNorm and NormFinder calculate mean expression values (M) and stability expression values (SV) which respectively take into account gene co-expression and inter-group variations in order to rank the candidate reference genes. The Cq values of the reference gene(s) validated by the three algorithms was used for expression data normalization (Cq_reference_) through obtaining ΔCq for each gene (ΔCq_target_ = Cq _target_–Cq_reference_). For gene expression comparisons within each lake, a replicate of an algae-grazer species was set as a calibrator sample and rest of the samples were normalized according to its ΔCq value (ΔΔCq_target_ = ΔCq_target_–ΔCq_calibrator_). In expression comparisons between the trophic niches across the lakes, the lowest expressed replicate for each target gene was used as a calibrator sample. Relative expression quantities (RQ) were calculated though E^−ΔΔ*Cq*^ method (Pfaffl, 2001) and their fold difference values (FD), after transformation of RQ values to logarithmic base 2 values, were used to perform statistical analysis (Bergkvist et al., 2010). The significant expression differences were determined using non-parametric Mann–Whitney *U* test (Wilcoxon rank sum test) (McKnight & Najab, 2010). We corrected the results for the within-lake comparisons using Benjamini–Hochberg multiple testing methods (Thissen et al., 2002) (Supplementary data 1). The effect sizes were also calculated for the within lake comparisons using Cohen’s *d* test (Cohen, 1992). Finally, to assess the species similarities in expression signature of the appetite regulating genes a dendrogram clustering was conducted using expression correlations calculated through Pearson correlation coefficients (r) using R version 3.5.1 (R Core Team, 2018).

## Results

### Validation of reference genes for expression analysis

The expression levels of candidate reference genes were variable; from the lowest expression level (highest Cq value) of *tbp* to the highest expression level (lowest Cq value) of *actb1* (Fig. 1B). Based on NormFinder, which takes into account the inter-group expression variations, *actb1*, *ef1a* and *rps11*, were ranked as the most stable genes in the brain of our study species from LM, LV and LT, respectively (Table 2). It should be noted that only *rps11* was always ranked among the top three genes across the lakes according to the NormFinder rankings. geNorm identified *actb1*, *ef1a* and *tuba1* as the most stable genes in LM, LV and LT, respectively. However, *rps11* appeared again to be the only gene ranked among the top three genes in all the lakes (ranked second in all the lakes) (Table 2). Finally, BestKeeper, which calculates expression stabilities through standard deviations in expression, ranked *rps11* as the most stable reference gene among the candidates in all the lakes (Table 2). Based on the findings by the three algorithms, *rps11* was found to have the most consistent expression stability, and therefore, its expression in the brain samples was selected as normalization factor (NF) for expression analyses of the appetite-regulating genes.

**Table 2:** Ranking and statistical analyses of reference genes in brain of six haplochromine species from three East African lakes.

	**BestKeeper**		**geNorm**		**NormFinder**	
	**Ranking**	**I**	**Ranking**	**M**	**Ranking**	**SV**
**Lake Malawi**	* rps11*	0.080	* actb1*	0.374	* actb1*	0.148
	*tuba1*	0.134	* rps11*	0.384	*hprt1*	0.176
	*rps18*	0.153	*tuba1*	0.392	* rps11*	0.210
	* actb1*	0.171	*hprt1*	0.400	*tuba1*	0.280
	*hprt1*	0.176	*rps18*	0.422	*rps18*	0.284
	*ef1a*	0.348	*ef1a*	0.491	*ef1a*	0.295
	*tbp*	0.349	*tbp*	0.577	*tbp*	0.519
	*gapdh*	0.935	*gapdh*	0.978	*gapdh*	1.168
**Lake Victoria**	* rps11*	0.076	ef1a	0.387	ef1a	0.228
	*actb1*	0.159	* rps11*	0.393	*actb1*	0.283
	*tbp*	0.167	*tbp*	0.403	* rps11*	0.295
	*ef1a*	0.194	*actb1*	0.408	*rps18*	0.386
	*hprt1*	0.204	*rps18*	0.429	*tbp*	0.413
	*rps18*	0.208	*hprt1*	0.490	*hprt1*	0.525
	*tuba1*	0.218	*tuba1*	0.516	*tuba1*	0.656
	*gapdh*	0.963	*gapdh*	1.298	*gapdh*	2.923
**Lake Tanganyika**	* rps11*	0.197	*tuba1*	0.535	* rps11*	0.033
	*actb1*	0.248	* rps11*	0.539	*rps18*	0.036
	*rps18*	0.257	*rps18*	0.549	*tbp*	0.087
	*tbp*	0.292	*tbp*	0.599	*tuba1*	0.138
	*tuba1*	0.300	*hprt1*	0.604	*actb1*	0.158
	*ef1a*	0.399	*ef1a*	0.643	*hprt1*	0.160
	*hprt1*	0.400	*actb1*	0.731	*ef1a*	0.386
	*gapdh*	1.867	*gapdh*	1.996	*gapdh*	4.896

**Notes.**

I BestKeeper index calculated through standard deviations in expression SV stability value M M value of stability

### Expression differences between herbivores and carnivores

At first, we used the relative expressions of all 16 target genes in each species in order to construct a dendrogram cluster representing the similarities between species in brain expression of appetite-regulating genes (Fig. 1C). The results showed that the similarities between the species are mainly determined by evolutionary relatedness by which species from the same lake (for Malawi or Victoria) are paired together. However, an interesting difference was observed for the LT species where the carnivore species (C.h) was clustered with the LM species and the herbivore species (P.p) branched distantly from the other clusters (Fig. 1C). This might indicate that the LT species with their much older evolutionary divergence have more distinct expression pattern of appetite-regulating genes prior to foraging, as outlined in more detail in the discussion. It also appears that the herbivore brain might have more diverged gene expression patterns for appetite-regulating genes in LT.

**Figure 2:**
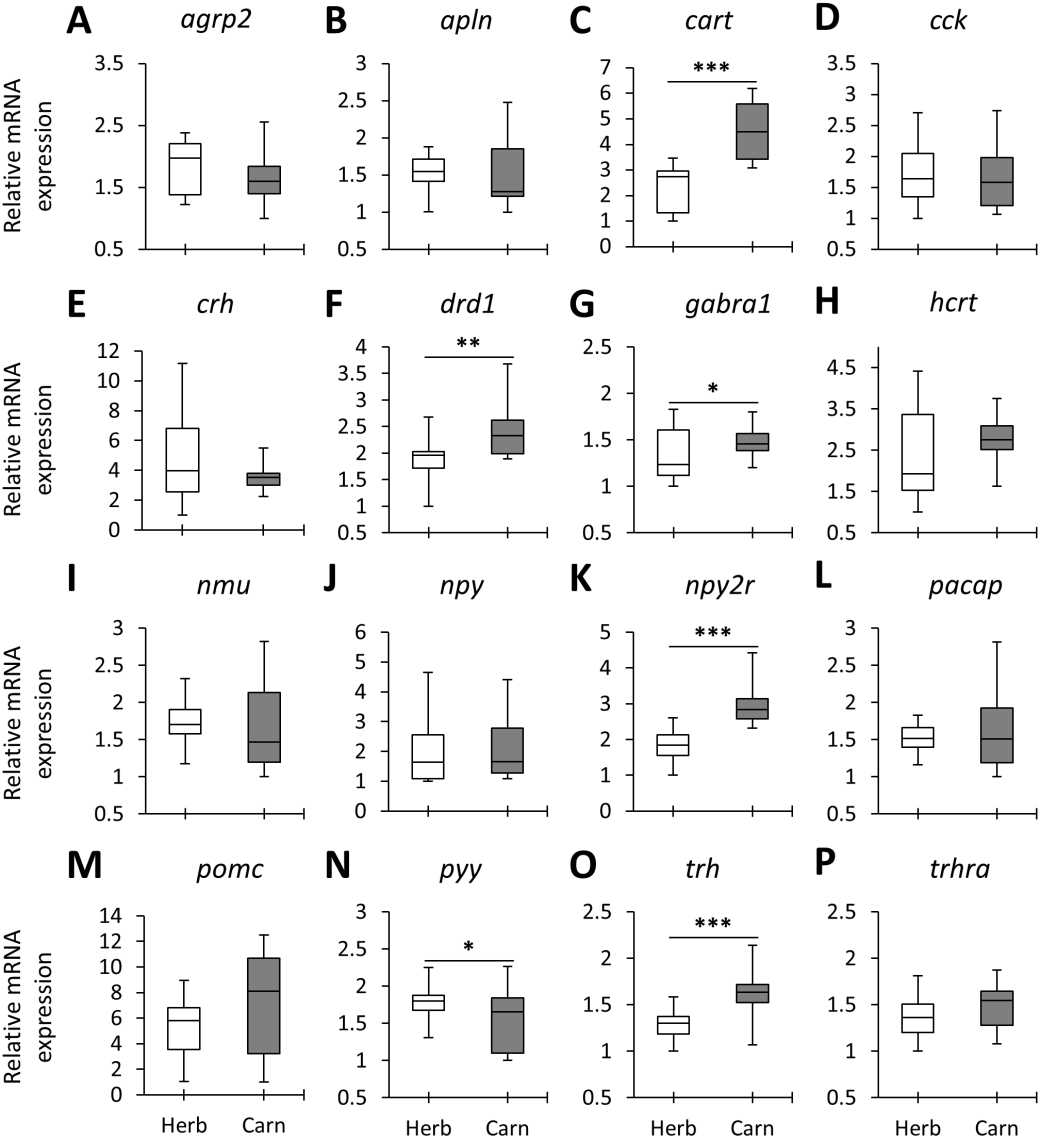
The herbivores versus carnivores expression differences of appetite-regulating genes in the brains of haplochromine cichlids at the end of the larval phase. (A–P) Comparisons of relative expression levels of 16 appetite-regulating genes in brain, all herbivore species from the three lakes combined versus all the carnivore species, at the end of larval development and prior to foraging. The statistical differences are shown by one, two and three asterisks above bars indicating *P* < 0.05, 0.01 and 0.001, respectively. The middle line in each box plot represents the median together with the 25/75 percentiles.

When the overall expression levels of the appetite-regulating genes were compared between herbivores and carnivores across the lakes six genes, *cart*, *drd1*, *gabra1*, *npy2r*, *pyy* and *trh* appeared to have differential expression (Fig. 2). Among these, *cart*, *gabra1* and *npy2r* displayed strong expression differences. Also, all of these genes, except *pyy*, had shown higher expression in the carnivores than herbivores (Fig. 2). These results demonstrate expression differences of certain appetite-regulating genes in herbivorous versus carnivorous haplochromine cichlids prior to initiation of their feeding. This also suggests that feeding behaviour can be already determined in the brain by differential expression of appetite-regulating genes before exposure to available food resources. However, considering the opposing appetite-regulating functions of these genes, i.e., *cart*, *drd1* and *npy2r* are anorexigenic whereas *gabra1* and *trh* are orexigenic genes (Table 1), it is difficult to interpret the feeding behaviour outcome of such contrasting expression differences.

**Figure 3:**
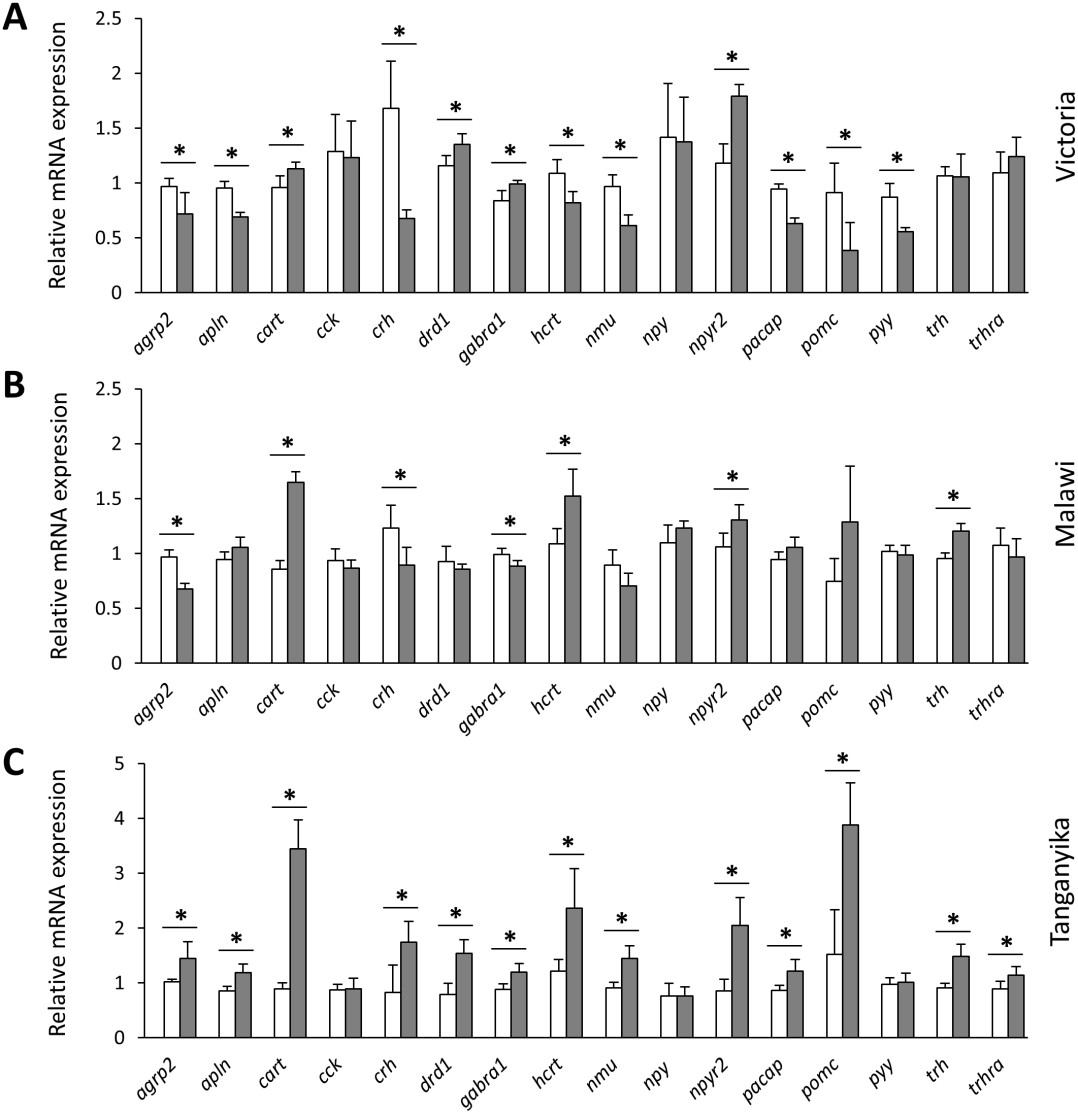
Within lake brain expression differences of appetite-regulating genes between herbivorous and carnivorous haplochromine cichlids at the end of the larval phase. (A–C) Comparisons of relative expression levels of 16 appetite-regulating genes in brain, between the herbivorous (white bars) and carnivorous (grey bars) species of each lake, at the end of the larval development and prior to foraging. The statistical differences are shown by asterisks above the bars indicating *P* < 0.05. Error bars represent standard deviations calculated from six biological replicates.

Next, we compared the expression levels of each gene between the herbivorous and carnivorous species within the lakes. All of the genes, except *cck* and *npy* , showed differential expression between the two trophic niches in at least one lake (Fig. 3). Out of the 16 tested genes, 7, 12 and 13 genes were differentially expressed in LM, LV and LT, respectively. The effect sizes, as indicated by the calculated Cohen’s *d* values (Supplementary data 1), further demonstrated that all the differentially expressed genes had large effect sizes (*d* values >0.8), indicating that the results have not been influenced by low statistical power. In LT, all of the 13 differentially expressed genes showed higher expression in the carnivore species, but this number declined by the age of divergence between the trophic niches in each lake, i.e., four out of the seven genes for LM and four out of the 12 genes for LV (Fig. 3). When comparing the lakes, four genes, *cart*, *hcrt*, *npy2r* and *trh*, showed similar expression difference between LT and LM, four genes, *agrp2*, *cart*, *crh* and *npy2r*, did between LT and LV, and four genes, *cart*, *drd1*, *gabra1* and *npy2r* between LM and LV. Among these, only *cart* and *npy2r* (both anorexigenic genes) had similar differences of higher expression level in carnivorous than herbivorous species in all the three lakes (Fig. 3). The differential expression of *cart* appeared to be increased in the carnivore brains according to the age of divergence between the contrasting species of each lake (i.e., LT >LM >LV). Moreover, *cart* displayed the strongest herbivorous versus carnivorous expression difference among the identified differentially expressed genes. The expression results of genes like *cart* and *npy2r* suggest that carnivory versus herbivory and possibly their related feeding behaviour in Haplochromine cichlids might be linked to divergence in brain expression of the anorexigenic genes prior to initiation of feeding.

## Discussion

Diversity in cichlid diet and foraging behaviors is thought to be a key factor facilitating their rapid divergence by enabling effective trophic specialization and ecological speciation (Liem, 1973). Plasticity in trophic morphology and physiology, manifested in jaw shape, intestine length and enzymatic activities, are believed to have played an important role in the adaptation to new habitats and the optimization of feeding during ontogeny (Sturmbauer, Mark & Dallinger, 1992; Takahashi & Koblmüller, 2011). But little is known about the link between the brain and foraging behavior. In particular, the appetite-regulating genes in the brain that might contribute to different dietary and swimming habits, prior to the onset of feeding have not been studied. Here we investigated the expression of appetite regulating genes in the brains of cichlids adapted to herbivorous and carnivorous trophic niches and identified two appetite-regulating genes, *cart* (or *cartpt1*) and *npy2r*, to have higher expression in the carnivore brains prior to the initiation of feeding across at least two of the lakes. *Cart* and *npy2r* genes are indicated to have anorexigenic function in both distant and closely related groups of fishes to cichlids (i.e., Cypriniformes and Perciformes, respectively) (Matsuda et al., 2012; Babichuk & Volkoff, 2013; Wang et al., 2014; He et al., 2015; Volkoff, 2016; Porter, Roberts & Maruska, 2017). This suggests potential involvement of the two anorexigenic genes in parallel trophic radiations of the cichlids used in this study. Furthermore, the increased expression of *cart* and *npy2r* in carnivorous species at the end of larval development can be reminiscent of the adult feeding behaviour based on which herbivorous species have an increased level of appetite compared to carnivorous species in order to compensate their nutritionally poorer diets. This may indicate heterochrony in brain expression of these genes prior to emergence of herbivorous versus carnivorous feeding behaviours during later ontogeny. Heterochrony in gene expression is already suggested as a potential molecular mechanism in divergent adaptive radiation of teleost fish (Albertson et al., 2010; O’Quin et al., 2011; Shkil & Smirnov, 2015).

The first gene, *cart*, or cocaine- and amphetamine-regulated transcript, encodes a pre-proprotein which proteolyzes to multiple active peptides and participates in biological processes related to regulation of appetite, energy balance, stress response, and reward and addiction responses (Volkoff, 2006; Volkoff, 2016; Koylu, Balkan & Pogun, 2006; Vicentic et al., 2007; Rogge et al., 2008). In most teleost fish including Perciformes, Salmoniformes and Gasterosteiformes only one *cart* isoform has been found (Murashita et al., 2009; Figueiredo-Silva et al., 2012; Striberny et al., 2015), whereas, in two model fish species; medaka and zebrafish (Beloniforme and Cypriniforme, respectively) more than one *cart* isoforms have been characterized (Murashita & Kurokawa, 2011; Akash et al., 2014). In a Haplochromine cichlid, *Astatotilapia burtoni*, six *cart* isoforms have been described and among them *cart*/*cartpt1* show the greatest similarity to mammalian *CART* gene (Hu et al., 2016). The brain expression pattern of *cart* appeared to be similar to its orthologs in other teleosts in the lateral posterior part of the hypothalamus (or lateral tuberal nucleus), which is also similar to the expression of mammalian *CART* in a comparable region called arcuate nucleus (Porter, Roberts & Maruska, 2017). Studies of Cypriniformes have demonstrated that *cart* induction inhibits food intake and increases locomotion and responsiveness to different sensory stimuli, and thus affecting feeding behavioural activity (Volkoff & Peter, 2000; Woods et al., 2014). It has been long known that predatory behaviour is directly influenced by the ability to respond to a range of sensory stimuli mediated by vision, olfaction and the lateral line in fish (Adams & Johnsen, 1986; Gehrke, 1988; Carr et al., 1996; Montgomery & Hamilton, 1997; Liao & Chang, 2003; Del Mar Palacios, Warren & McCormick, 2016). In addition, the decrease in brain expression of anorexigenic genes has been linked to the transition from carnivory to herbivory feeding behaviour in grass carp (He et al., 2015).

In our study, the increased *cart* expression in the carnivore brains prior to feeding may indicate less appetite and a predisposition for more environmental responsiveness in the carnivores, which may be a favorable behaviour for predatory-based trophic specialization. Furthermore, the conserved anorexigenic role of CART peptides in teleost fish has been demonstrated in a wide range of species during fasting and re-feeding experiments (reviewed in Volkoff, 2016). Interestingly, we found that the difference in *cart* expression level between the herbivorous and carnivorous species in each lake to be associated with the age of divergence in each lake, i.e., the older divergence had the highest difference in *cart* expression levels (Fig. 3). In the youngest lake (LV), on the other hand, only slight expression difference between the herbivorous and carnivorous species was found. This is especially interesting as the cichlids from older lakes have longer larval developmental periods as they have larger yolk sacs that provide nourishment for longer, so food intake may need to be inhibited for longer (Dreo and Gallaun, 2018, unpublished data). We also found the similar pattern of increased carnivorous expression in the two older lakes for *trh*, thyrotropin-releasing hormone, which is characterized as orexigenic factor in Cypriniformes through increasing feeding and locomotor behaviours (He et al., 2015; Volkoff, 2016). However, such a function has not been identified in Cichliformes or Perciformes, and its induced expression in carnivorous species of LT and LM can be the result of strong induction of *cart* in these species. This is due to the fact that *trh* is at downstream of *cart* in vertebrates and it can function as negative regulatory feedback for *cart* signal (Fekete & Lechan, 2006). Based on this negative feedback, prolonged expression of the anorexigenic *cart* gene induces the transcription of orexigenic *trh* which in turn can control *cart* expression through a complex molecular cascade (Fekete & Lechan, 2006). Therefore, the increased expression of *trh* in carnivorous species can be in fact a confirmation for functional activation of *cart* signal in their brain.

The second gene, *npy2r*, encodes a receptor of Neuropeptide Y (*npy*), and interestingly, an ortholog of the same receptor has been identified to have reduced expression during the transition from carnivory to herbivory in grass carp (He et al., 2015). The ligand of this receptor, *npy*, is expressed in different tissues, particularly in brain and intestine, and its encoded peptide (NPY) has been one of the first studied appetite-regulating factors in fish (Volkoff, 2016). In this study we found reduced expression of *npy2r* in the brain of herbivores which is consistent with the suggested anorexigenic role of *npy2r* in grass carp (He et al., 2015). Although, the ligand of *npy2r*, NPY peptide, acts as an orexigenic factor in most teleost fish species (reviewed in (Volkoff, 2016), *npy2r* is among the NPY receptors in vertebrates that functions as inhibitory auto-receptor, and thus playing an opposite role to NPY in appetite regulation (Chen et al., 1997; Naveilhan et al., 1999).

Overall, most of the selected appetite-regulating genes showed no consistent expression differences between herbivores and carnivores across the three lakes indicating that most of these genes probably do not contribute to determination of feeding behaviour prior to foraging in the selected cichlid species. Moreover, their expression differences between the two trophic niches showed the most discrepancies between the species of the youngest and oldest lake adaptive radiations (LV versus LT). This might reflect more extensive divergence in evolutionary trajectories of the LV and LT lineages in expression of the appetite-regulating genes. Although, the consistently increased expression of the two anorexigenic genes, *cart* and *npy2r*, in carnivores in all the lakes could imply on their potential role in determination of the feeding behaviours prior to foraging in the cichlid species in this study. Further functional investigations are required to confirm such a role for appetite regulating genes in fish. The number of species used in this study to represent each trophic niche is not sufficient to conclude that signals mediated by *cart* and *npy2r* are potential drivers for divergent trophic specialization across the entire Haplochromini tribe. In addition, it is not clear if the peptides encoded by these genes interact with other appetite-regulating factors and whether they override the effects of the other differentially expressed factors in the brain.

Other behavioural factors that can affect or being affected by gene expression in the brain are the divergent habitat use and swimming strategies by species with contrasting trophic specialization. Although, all the species in this study (except *C. horei*) are reported to occupy somewhat similar habitats in the three lakes: *M. lutea*; rock-dwelling (Seehausen et al., 1998), *H. thereuterion*; rock-dwelling with surface oriented swimming (Seehausen, 1996), *S. fryeri*; rocky and intermediate habitats (Konings, 1993), *P. sp. chitimba*; rock-dwelling (Johnson et al., 2019), *P. polyodon*; rock-dwelling (Kohda, 1998), and *C.horei*; rocky-sandy or muddy and sediment-rich habitats (Wanek & Sturmbauer, 2015). The difference in swimming strategies, however, can be more consistent between the two trophic niches, i.e., carnivorous species expected to have more surface-oriented or free-range swimming in the water column, and it is already known that most appetite-regulating genes can affect swimming, locomotor activity and food searching behaviour in fish (Volkoff, 2016). It is worth noting that the observed expression differences could be originated from proportional anatomical discrepancies in the brain regions expressing these genes and not necessarily the result of transcriptional changes. This is particularly important topic for future histological investigations, since anatomical variations in brain regions have been already demonstrated between East African cichlids with divergent ecological adaptation (Sylvester et al., 2010). Finally, we should emphasize that the time to reach to stage 26 for haplochromine cichlids varies between the lakes, i.e., the length of this time is positively correlated with the age of the lakes, and therefore, this limits such gene expression studies to only comparisons of the species within each lake .

## Conclusions

Diet is a major factor mediating adaptive divergence in the adaptive radiation of cichlids fishes. Here we present the first step towards delineating the genes involved in regulating appetite in herbivorous and carnivorous cichlids prior to the onset of independent feeding. We identified two anorexigenic genes, *cart* and *npy2r*, to be differentially expressed between the two trophic categories in parallel cichlid radiations of three East African Lakes, which is suggestive of their role in controlling satiety in these species. It might also imply that appetite gene regulation is genetically hardwired. However, future studies investigating potential links between the functions of appetite-regulating genes and specific foraging tactics are essential to conclude that these results advocate low plasticity in regard with herbivory and carnivory in cichlids. Also, large-scale studies using transcriptomic approach with more species (representing carnivorous and herbivorous trophic niches) from each lake are required to test whether appetite-regulating genes are among the divergent genetic signals in brain participating in trophic niche specialization during cichlid adaptive radiation. In conclusion, we present a first glimpse into an important aspect of feeding in cichlids that is the regulatory control of appetite in the context of contrasting trophic niche adaptation.

## List of abbreviations

LT: Lake Tanganyika
LM: Lake Malawi
LV: Lake Victoria

## Supplemental Information

Table S1 Information about qPCR primers used in this study

Data S1 Raw Cq values

### Additional Information and Declarations

#### Data Availability

The following information was supplied regarding data availability: All data generated or analysed are available in the Results section and in Supplementary Files.
